# Proteomic Landscape of Adeno-Associated Virus (AAV)-Producing HEK293 Cells

**DOI:** 10.3390/ijms222111499

**Published:** 2021-10-25

**Authors:** Lisa Strasser, Stefano Boi, Felipe Guapo, Nicholas Donohue, Niall Barron, Alana Rainbow-Fletcher, Jonathan Bones

**Affiliations:** 1 NIBRT—National Institute for Bioprocessing Research and Training, Foster Avenue, Blackrock, A94 X099 Dublin, Ireland; lisa.strasser@nibrt.ie (L.S.); stefano.boi@nibrt.ie (S.B.); felipe.guapo@nibrt.ie (F.G.); Nicholas.Donohue@nibrt.ie (N.D.); niall.barron@nibrt.ie (N.B.); 2 School of Chemical and Bioprocess Engineering, University College Dublin, Belfield, D04 V1W8 Dublin, Ireland; 3 Pharmaron, 12 Estuary Banks, Speke, Liverpool L24 8RB, UK; Alana.RainbowRobinson@pharmaron-uk.com

**Keywords:** proteomics, LC-MS/MS, SP3, label-free quantitation, HEK293 cells, adeno-associated virus (AAV), gene therapy

## Abstract

Adeno-associated viral (AAV) vectors are widely used for gene therapy, providing treatment for diseases caused by absent or defective genes. Despite the success of gene therapy, AAV manufacturing is still challenging, with production yields being limited. With increased patient demand, improvements in host cell productivity through various engineering strategies will be necessary. Here, we study the host cell proteome of AAV5-producing HEK293 cells using reversed phase nano-liquid chromatography and tandem mass spectrometry (RPLC-MS/MS). Relative label-free quantitation (LFQ) was performed, allowing a comparison of transfected vs. untransfected cells. Gene ontology enrichment and pathway analysis revealed differential expression of proteins involved in fundamental cellular processes such as metabolism, proliferation, and cell death. Furthermore, changes in expression of proteins involved in endocytosis and lysosomal degradation were observed. Our data provides highly valuable insights into cellular mechanisms involved during recombinant AAV production by HEK293 cells, thus potentially enabling further improvements of gene therapy product manufacturing.

## 1. Introduction

Gene therapy using adeno-associated viral (AAV) vector delivery is a rapidly evolving field of the biotherapeutics industry. While the first recombinant AAV product, Glybera, was approved only in 2012, there are now over 150 clinical trials involving AAV registered on ClinicalTrials.gov [1].

However, despite this clinical activity, production of AAV-based therapeutics faces several significant challenges. Among others, viral particle yield is low, resulting in high manufacturing costs. In fact, Zolgensma, which is used for treating spinal muscular atrophy, is still the most expensive biotherapeutic product on the market, with a cost close to USD 2 million per treatment. Various approaches to address limited production yields have been investigated, with most aiming towards improved transfection of the production cells, either by optimizing one parameter at a time or via design-of-experiment (DOE) methodologies [2,3]. Although such approaches have shown to increase recombinant AAV (rAAV) titers, these studies did not investigate how rAAV production affects endogenous host cell protein expression and how host cell signaling might be used to further enhance productivity levels.

Proteomic profiling provides important insights into underlying cellular mechanisms involved in recombinant protein expression. In recent years, proteomics has been extensively used to study, for example, Chinese hamster ovary (CHO) cells, which are routinely used to produce monoclonal antibodies (mAbs) [4,5]. Using the outputs of these proteomic investigations, CHO cells have been actively engineered, and bioprocessing conditions optimized, in order to modify the cell cycle and energy metabolism of the cells, aiming towards increased levels of specific productivity [6,7,8,9]. Despite application of proteomics to study recombinant protein production in mammalian cells, there is very limited information about differential expression and cell signaling involved in rAAV expression. In 2014, Dong et al. studied copurifying host cell proteins in AAV vector preparations [10], using gel electrophoresis combined with liquid chromatography–mass spectrometry. They concluded that some copurifying proteins are involvement in the AAV life cycle. Proteomic analysis was performed by Aponte-Ubillus and co-workers to study cellular rAAV production in yeast, and they reported differential expression of proteins involved in protein folding, cell metabolism, and cellular stress responses. They subsequently used their observations for targeted overexpression of various proteins, which resulted in increased vector yields [11]. However, the most widely used expression systems for rAAV are HEK293 and Sf9 cells [1]. Although HEK293 cells have been previously studied while producing virus-like particles [12], there is still no detailed information on how rAAV expression affects cellular signaling.

In this study, HEK293 cells were used to investigate the effects of recombinant AAV production on the host cell proteome. Using standard triple transfection, AAV serotype 5 (AAV5) expressing GFP under the control of a CMV promotor was produced. Seventy-two hours post-transfection, cell lysates and cell culture supernatants were harvested and used for proteomic profiling. A semi-automated version of the SP3 protocol [13] for sample preparation was employed, followed by proteomic profiling via reversed phase nano-liquid chromatography and tandem mass spectrometry (RPLC-MS/MS). A total of 6534 protein groups were identified and label-free quantification (LFQ) of 4996 proteins was possible. Gene ontology enrichment analysis provided important insights into affected cellular processes upon rAAV production by the cells. Automated handling of magnetic beads during sample preparation yielded excellent reproducibility, while the use of state-of-the-art instrumentation allowed for in-depth profiling without the necessity of sample pre-fractionation.

The data presented here provides interesting insights into cellular mechanisms involved during rAAV expression and could help identifying strategies for further improvements of AAV manufacturing.

## 2. Materials and Methods

### 2.1. Cell Culture

#### 2.1.1. Plasmids and Cells

FreeStyle 293-F cells (Thermo Fisher Scientific, Waltham, MA, USA, Cat. R79007) were maintained in suspension in BalanCD HEK293 (Cat. 91165-1L, FUJIFILM Irvine Scientific, Wicklow, Ireland) chemically defined, serum-free medium supplemented with 4 mM L-Glutamine. The cells were cultured in 125 mL Erlenmeyer flasks at 37 °C, 8% CO_2_, and agitated at 170 rpm with a humidified atmosphere. The plasmids used for triple transfection were: pAdDeltaF6 (gift from James M. Wilson, Addgene plasmid # 112867), pAAV2/5 (gift from Melina Fan, Addgene, Watertown, MA, USA, plasmid # 104964), and pAAV-GFP (gift from John T. Gray, Addgene plasmid # 32395).

#### 2.1.2. Triple Transfection

FreeStyle 293-F cells were seeded in 4.5 mL medium at 1 × 10^6^ cells/mL in 50 mL bioreactor tubes, incubated overnight at 37 °C, 8% CO_2_, and agitated at 170 rpm with a humidified atmosphere. On the following day, cells were triple transfected with 3 µg/mL of pAAV-GFP, pAAV2/5, and pAdDeltaF6 (molar ratio 1:1:1) and 1:2 ratio of Polyethylenimine MAX (PEI “MAX”, Polysciences, Warrington, PA, USA). The three plasmids and the PEI-MAX reagent were diluted in two separate tubes containing 250 µL of medium, and the contents of the two tubes were mixed and incubated for 15 min at room temperature prior to being added to the cells. Transfections were performed in triplicate. Additionally, three samples were mock-transfected by adding 500 µL of fresh cell culture medium for use as a negative control. Then, 72 h post-transfection, cell viability was measured by trypan blue staining on the LUNA-II Automated Cell Counter (Logos Biosystems, Villeneuve d’Ascq, France), while GFP fluorescence was detected on an EVOS M5000 Imaging System (Thermo Fisher Scientific). Following this, 5 mL of each culture were collected and centrifuged at 16,000× *g* for 20 min, and the supernatants were aliquoted and stored at −80 °C for further analysis, while the cell pellets were resuspended in 1.25 mL of medium. Then, 500 µL of the resuspended cell pellets were washed with PBS and processed for proteomic analysis, while the remaining 750 µL were processed by 3 liquid nitrogen/37 °C bath freeze/thaw cycles. After the last thaw, MgCl_2_ and Benzonase (Merck, Darmstadt, Germany) were added to the samples to a final concentration of 2 mM and 100 U/mL, respectively. The samples were then incubated at 37 °C for 1 h. After incubation, the samples were centrifuged at 16,000 × *g* for 20 min and the supernatants were stored at −80 °C until further analysis.

#### 2.1.3. AAV qPCR Titration

AAV viral genome titer was determined in the supernatant and cell pellet samples by qPCR. First, 10 µL of each sample was treated with 1.25 U/µL Benzonase (Merck), followed by proteinase K (Thermo Fisher Scientific, Dublin, Ireland) digestion using 6-10 U/mL. Two dilutions of the digested sample (1:100 and 1:1000) were used for qPCR, performed using Fast SYBR Green Master Mix (Applied Biosystems, Carlsbad, CA, USA) and primers specific to the AAV2 ITRs flanking the GFP expression cassette in the pAAV-GFP plasmid. qPCR was performed using a QuantStudio 3 Real-Time PCR System (Thermo Fisher Scientific), and the viral genome titers were calculated using the QuantStudio design and analysis software (version 1.5.1) based on the generated standard curve.

### 2.2. Sample Preparation for Proteome Analysis

For proteome analysis, samples were prepared using a semi-automated version of the SP3 protocol [13]. Cell pellets, containing approximately 7 × 10^6^ cells, were lysed using 1 × RIPA buffer (Cell Signaling Technology, Dublin, Ireland) containing 1 × protease inhibitor (cOmplete™, Mini, EDTA-free Protease Inhibitor Cocktail, Sigma, Wicklow, Ireland) followed by sonication 4 times for 30 s using a Fisherbrand Model 50 Sonic Dismembrator (Fisher Scientific, Dublin, Ireland) set to 20.0 kHz. Cell debris were removed by centrifugation at 14,000× *g* for 10 min. Proteins from supernatant samples were precipitated via acetone precipitation at −20 °C overnight. After centrifugation at 14,000× *g* for 10 min, protein pellets were dried at room temperature and then dissolved in PBS with 1 × protease inhibitor. Protein concentration of resulting samples was determined using the Pierce™ 660 nm Protein Assay Kit (Thermo Fisher Scientific, Dublin, Ireland) as instructed by the manufacturer. Afterwards, aliquots containing 50 µg of protein in 95 µL RIPA buffer or PBS, respectively, were prepared and used for further steps. Prior to tryptic digestion, proteins were reduced via incubation at 56 °C for 30 min using 5 mM 1,4-dithiothreitol and alkylated with 10 mM iodoacetamide for 30 min at room temperature in the dark. Then, 50% (*v*/*v*) ethanol was added to the samples.

The following steps were carried out using a KingFisher Duo Prime purification system under the control of Thermo Scientific BindIt software version 4.0. For each sample, 10 µL of a 1:1 mixture of Sera-Mag SpeedBeads (P/N 45152105050250 and 65152105050250 from Cytiva, Buckinghamshire, UK) were prepared by washing the beads 3 times with 500 µL LC-MS grade water (Fisher Scientific), using medium mixing to prevent sedimentation of the beads. After each step, beads were recollected for 4 × 10 s. Following preparation, the carboxylate-modified magnetic beads were added to the cell lysate as well as supernatant samples. Proteins were bound to the beads during a 10 min incubation, followed by 3 wash steps using 80% ethanol. During each wash step, samples were mixed at medium speed for 2 min followed by recollection of the beads for 4 × 10 s. After this purification, proteins were released from the beads and digested by adding the beads into 50 mM ammonium bicarbonate (Sigma) containing 1 µg trypsin (Promega, Madison, WI, USA). Proteins were digested for 4 h, using medium mixing at 37 °C. Following digestion, magnetic beads were removed, samples were acidified by adding 0.1% formic acid (*v*/*v*) and then directly used for analysis via LC-MS as described below.

### 2.3. Reversed Phase Liquid Chromatography-Tandem Mass Spectrometry (RPLC-MS/MS)

Analysis of biological replicates was performed in duplicate. All spectra were acquired using a Q Exactive™ Plus Hybrid Quadrupole-Orbitrap™ mass spectrometer (Thermo Fisher Scientific, Bremen, Germany) coupled to an UltiMate 3000 RSLCnano HPLC system by means of an EASY-Spray source (Thermo Fisher Scientific, Germering, Germany). We loaded 1 µg per sample onto a C18 Nano-Trap Column, followed by separation using an EASY-Spray Acclaim PepMap 100, 75 µm × 50 cm column (Thermo Fisher Scientific, Sunnyvale, CA, United States) maintained at 45.0 °C at a flow rate of 250.0 nL/min. Separation was achieved using a gradient of (A) 0.10% (*v*/*v*) formic acid in water and (B) 0.10% (*v*/*v*) formic acid in acetonitrile (LC-MS optima, Fisher Scientific). Gradient conditions were as follows: 2% B for 3 min, followed by an increase to 5% within 5 min. Then, a linear gradient of 5–15% in 112 min, followed by another increase to 25% B in 60 min and a step to 35% B in 5 min was used. The separation was followed by 2 wash steps at 80% B for 5 min, and the column was re-equilibrated at 2% B for 15 min.

MS analysis was performed in positive ion mode. Full scans were acquired at a resolution setting of 70,000 at *m*/*z* 200 with a scan range of *m*/*z* 300–2000 using an automatic gain control (AGC) target of 1 × 10^6^ with a maximum injection time (IT) of 100 ms. The 15 most intense precursors ions were selected for HCD fragmentation using a normalized collision energy setting of 29. Fragment scans were acquired at a resolution setting of 17,500 with an AGC target of 1e5 and 100 ms max IT. For isolation of precursor ions, an isolation window of 1.2 *m*/*z* with 10 ppm tolerance was used. An intensity threshold of 8e4 was used, while unassigned charge states as well as charges >8 were excluded. The dynamic exclusion time was set to 60 s.

### 2.4. Data Analysis

Raw files were analyzed using Thermo Scientific Proteome Discoverer 2.3. MS/MS spectra were searched against a human database (Uniprot, downloaded 24th of February 2021) using Sequest HT. For the protein identification and label-free quantitation (LFQ), mass tolerances of 10 ppm for precursor ions and 0.6 Da for fragment ions were allowed. Furthermore, trypsin was set as enzyme with a maximum of 2 missed cleavages. Search criteria included carbamidomethylation of cysteines as static modification, as well as oxidation and N-terminal acetylation as dynamic modifications. Normalization of LFQ values was performed based on total peptide amounts.

Further statistical analysis was performed in Perseus version 1.6.6. Significance of log2(x)-transformed LFQ values was determined using volcano plots employing a two-sided *t*-test with a false discovery rate of 0.05, a variance parameter (S0) of 0.01, and a minimum fold change of 1.5. For biological interpretation, a gene ontology (GO) enrichment analysis was performed using a Fisher’s exact test (*p* ≤ 0.05). Pathway analysis was performed using QIAGEN’s Ingenuity Pathway Analysis software (IPA, QIAGEN Inc., Redwood City, CA, USA, https://www.qiagenbioinformatics.com/products/ingenuitypathway-analysis, accessed on 20 April 2021).

### 2.5. Chloroquine Treatment

To study the effect of a perturbed endocytic pathway on AAV titers, HEK293 cells were treated with chloroquine (CQ, P/N 11416331, Fisher Scientific, Dublin, Ireland). Therefore, cells were cultured and transfected as described in Section 2.1.2. Six hours after triple-transfection, cells were treated with 5 and 25 µM CQ, respectively. Additionally, a positive control (no CQ treatment) as well as a negative control (only using the plasmid pAAV-GFP) were prepared. For each condition, biological duplicates were prepared. Cell and supernatants were harvested 72 h post-transfection, followed by cell viability assessment using trypan blue staining (data not shown) and AAV qPCR titration as described before (Section 2.1.3).

## 3. Results and Discussion

### 3.1. Cell Viability and AAV Production

HEK293 cells maintained in suspension were either transfected for AAV5 expression via triple transfection or mock transfected as a negative control. For both conditions, biological triplicates were prepared. Seventy-two hours after transfection, cells were counted, viability was assessed using trypan blue exclusion, and GFP fluorescence was detected. Cells were then harvested to determine the AAV viral genome titer using qPCR, and samples were processed for proteomic analysis.

**Figure 1:**
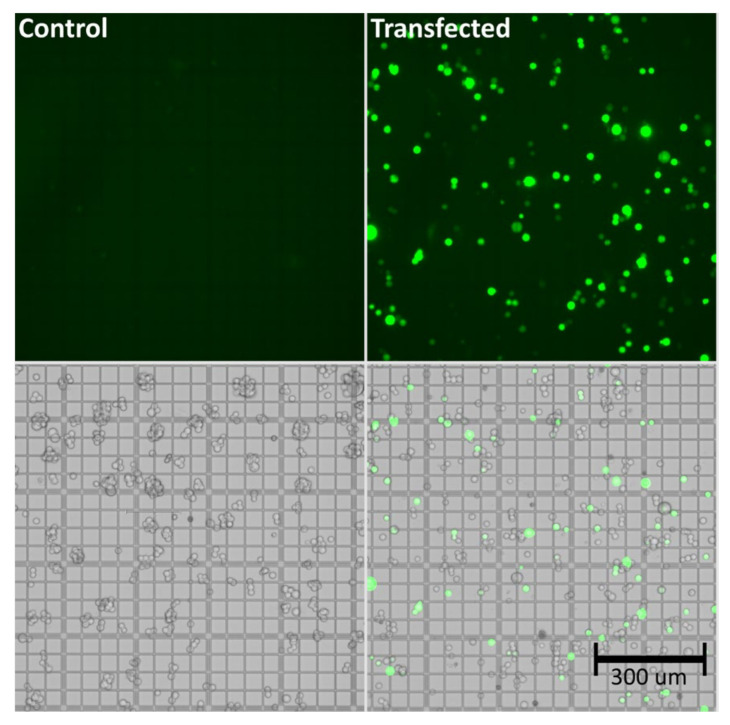
Bright-field and fluorescence images of untransfected (control) and transfected (GFP-expressing HEK293) cells at 72 h post transfection. The cells were transfected with 3 µg/mL of pAAV-GFP, pAAV2/5, and pAdDeltaF6 (molar ratio 1:1:1), and a 1:2 ratio of PEI “MAX.

As shown in Table 1, viability of the cells was maintained at high levels (>90%) throughout the experiment, even following triple transfection. The total cell count was at a similar level comparing transfected vs. control groups. Successful transfection was demonstrated via determination of the viral genome titer, which was found to be approximately 1.0 × 10^10^ viral genomes per mL (vg/mL), as well as by the amount of GFP positive cells (>60%). Effective transfection, indicated by GFP expression, was also demonstrated based on the bright-field and fluorescence images shown in Figure 1.

### 3.2. Proteomic Profiling

**Figure 2:**
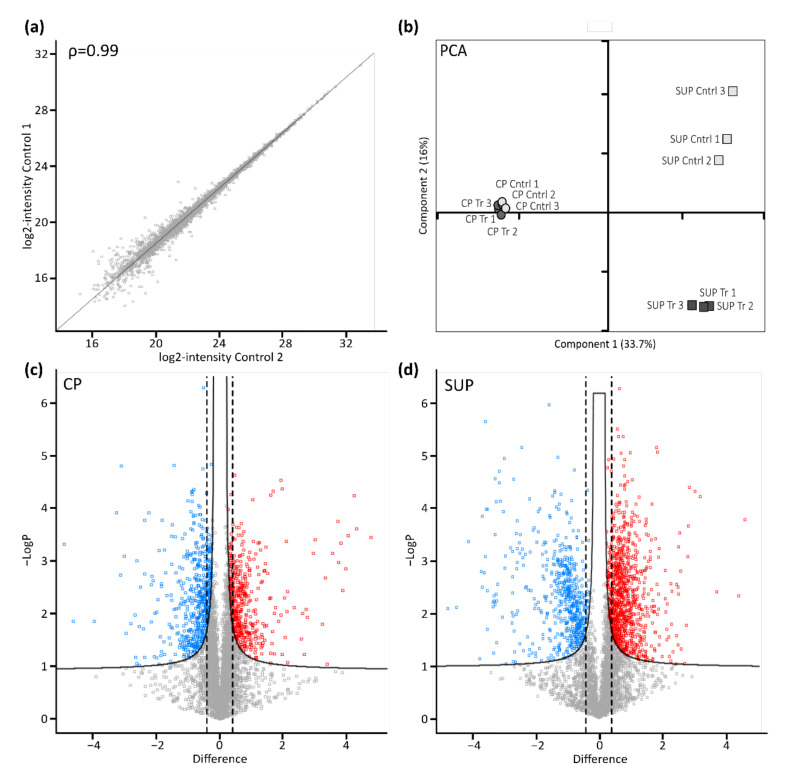
Proteomic profiling of AAV expressing HEK293 cells. (**a**) Scatter plot shows correlation between biological replicates. Shown are log-transformed intensity values of cell pellet control samples 1 vs. 2 with a Pearson correlation of ρ = 0.99. (**b**) Principal component analysis (PCA) shows relationship between biological replicates (n = 3) and treatment condition (control: light grey; transfected: dark grey) for cell pellets (CP, circles) and cell culture supernatant (SUP, squares). (**c**,**d**) Transfected vs. untransfected cell pellet (CP) and supernatant (SUP) samples were compared using volcano plots with cut-off curves indicating significance determined by a two-sided *t*-test with a permutation-based FDR of 0.05 and S0 of 0.1 (n = 3). Additionally, a minimum fold change (= log2-transformed difference) of 1.5 was set as threshold for significance as indicated by the dashed lines. Significantly downregulated proteins are indicated in blue, upregulated proteins are shown in red.

Following cell harvest, cell pellets (CP) and supernatants (SUP) were prepared for proteome analysis using a modified version of the SP3 protocol. As indicated for control samples by the scatter plot in Figure 2a, automatic handling of magnetic beads used for sample preparation in combination with high resolution LC-MS instrumentation yielded excellent reproducibility. Generally, duplicate analysis of biological replicates resulted in a Pearson correlation coefficient of >0.99. Following filtering of the data for a minimum of two unique peptides per protein and 70% valid values per group, remaining missing values were imputed based on a standard distribution. Further statistical analysis by means of principal component analysis (PCA, Figure 2b) revealed a clear differentiation between transfected and control samples. Interestingly, the effect was more evident in SUP samples, which might be due to differences in the complexity of those samples. Nevertheless, sample groups derived from CP also clearly segregated, especially when analyzed separately (not shown), while biological replicates clustered, again demonstrating high reproducibility.

To further assess significantly differentially expressed proteins upon transfection in CP (Figure 2c), as well as SUP samples (Figure 2d), a two-sided *t*-test with a permutation-based false discovery rate (FDR) of 0.05 was applied and plotted as volcano plots. Furthermore, a minimum fold change of 1.5 was set as the significance threshold. Thereby, 530 proteins in CP samples and 1275 proteins in SUP samples were found to be significantly differentially expressed upon transfection for AAV5 production. A complete list of identified and significant proteins can be found in the supplementary excel file.

Interestingly, the strongest upregulation was observed for proteins involved in cell organization and biogenesis, while the most intense downregulation of proteins was detected for proteins participating in metabolic processes (see Supplementary Materials). For example, the Ena/VASP-like protein (EVL) was found to be highly upregulated in AAV5-producing cells. EVL is known to control filament elongation, thus regulating actin dynamics [14]. In recent years, it was shown that cytoskeleton dynamics play a pivotal role for several cellular processes, requiring rigid control by, among others, Rho-GTPases. Cytoskeleton rearrangement upon Rho-GTPase signaling was shown to be essential not only for cell migration and cytokinesis or morphogenesis but also for endo/exocytosis [15]. Endo- and exocytosis are interesting in the context of AAV transduction. As Berry et al. described, AAV uses various mechanisms for endocytosis during cellular uptake [16]. During rAAV production, released particles might infect surrounding host cells, resulting in an activation of endocytosis-related pathways.

Another noteworthy observation is the strong upregulation of MAN2A2 observed in CP samples of transfected cells. MAN2A2 is an alpha-mannosidase involved in golgi-trafficking and, importantly, N-glycosylation of proteins [17]. While glycosylation is more often discussed in relation with AAV transduction [18], it was recently shown that capsid proteins (VPs) of AAV8 can be glycosylated [19]. This is of particular interest, as glycosylation is a critical quality attribute of biotherapeutics, requiring close monitoring [20]. While glycosylation of the AAV5 produced by HEK293 cells under investigation here would require further examination, higher expression levels of certain mannosidases are potentially interesting to monitor as they might be used for targeted modulation of certain post-translational modifications (PTMs) in AAV gene therapy products.

Notably, there are several proteins involved in cell cycle control and cell division which were found to be strongly downregulated in transfected cells, such as Aurora kinase A (AURKA). AURKA is a mitotic serine/threonine kinase which plays an important role in the regulation of cell cycle progression [21]. Hence, downregulation of AURKA could implicate an impacted cell cycle, resulting in reduced proliferation. While in the present study the viable cell count was not reduced 72 h post-transfection, it is important to note that harvest times might vary depending on processing conditions and maintaining high viability as well as proliferation throughout the production process is essential.

Generally, proteins that were highly up- or downregulated upon host cell transfection might represent potential biomarkers that can either be used for maintaining ideal cell culture conditions or possibly for targeted engineering of the cells aiming for higher specific productivity.

#### 3.2.1. Gene Ontology Enrichment Analysis

**Figure 3:**
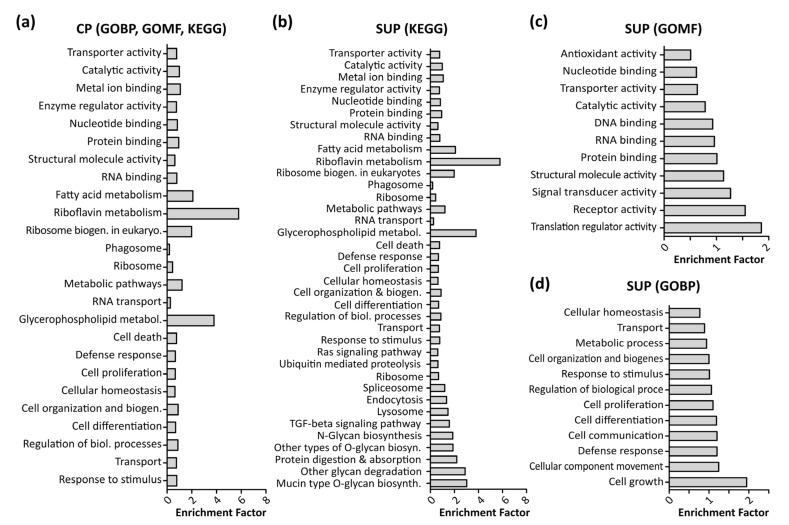
Results of gene ontology (GO) enrichment analysis using a Fisher’s exact test with a *p*-value threshold of 0.05 performed in Perseus (version 1.6.6). Shown are enriched Kegg pathways, GO molecular functions (GOMF), and GO biological processes (GOBP) in cell pellet samples (CP, (**a**)) as well as supernatant samples (SUP, **b**–**d**).

To enable further biological interpretation of the data, differentially expressed proteins were analyzed using gene ontology (GO) enrichment analysis employing a Fisher’s exact test with a *p*-value threshold of 0.05. Figure 3 shows significantly enriched GO terms in CP samples (a) as well as SUP samples, grouped in KEGG signaling pathways (b), molecular function (GOMF, c), and biological processes (GOBP, d). Notably, due to the higher amount of differentially expressed proteins detected, analysis of SUP samples revealed a higher number of enriched GO terms. A complete list of enriched GO terms can be found in the supplementary excel file. 

In line with previous findings, fundamental processes such as cell growth, proliferation, and differentiation, and defense response mechanisms were found to be enriched [22]. Additionally, as expected upon transfection, translational processes such as RNA and DNA binding, translational regulator activity, and ribosome biogenesis were significantly enriched.

Interestingly, riboflavin as well as glycerophospholipid metabolism were found to be highly enriched in CP and SUP samples. Riboflavin metabolism is known to be involved in mitochondrial beta-oxidation, and is therefore essential for energy production within the cells [23]. Apart from the necessity of energy production to maintain cell viability, it has also been shown that energy metabolism plays an important role during recombinant protein expression [5]. In the past, cellular production systems have been greatly engineered in order to modify cell metabolism, aiming towards higher specific productivity rather than biomass production [24].

**Figure 4:**
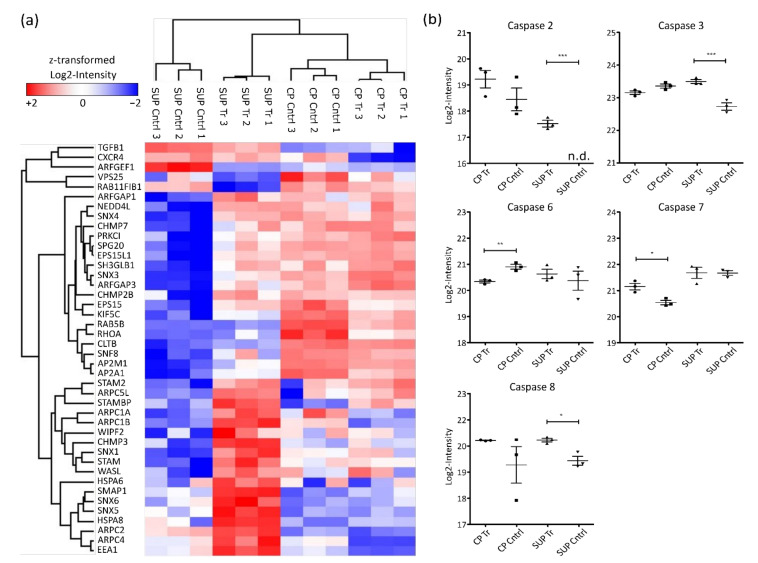
Hierarchical clustering of significantly differentially expressed proteins involved in endocytosis (**a**) based on Euclidian distance. Shown are log2-intensities after z-transformation. Red indicates upregulation while blue shows downregulation of the corresponding proteins. (**b**) Log2-transformed LFQ intensity of identified caspases. Significance was determined using an ANOVA with a Tukey’s post-test (*** *p* ≤ 0.001; ** *p* ≤ 0.01; * *p* ≤ 0.05; n = 3).

Glycerophospholipids are important components of cellular membranes which require tight regulation to preserve the cell’s ability for biosynthesis, intracellular trafficking, and degradation. Although limited information is available, it is known that upon AAV uptake by cells, endosomal trafficking and especially acidification play an important role for infectivity [25,26]. Enrichment of glycerophospholipid metabolism in the present study might be induced by two processes, either AAV transport upon expression by the host cells or via AAV reinfection of cells, leading to internal processing. This observation would also align with the enrichment of endocytosis and lysosomal proteins. Generally, as depicted in the heatmap in Figure 4a, endosomal proteins were found to be highly abundant in SUP samples of transfected cells.

As mentioned above, also strong downregulation of proteins involved in cell cycle control and cell division was observed. Correspondingly, cell death was found to be enriched in AAV5-producing HEK293 cells. Most prominent regulators of cell death are caspases, a family of aspartate-specific cysteine proteases known to be essential for initiation and execution of apoptosis [27]. In the presented dataset, 5 different caspases were quantified (Figure 4b). Among them, CASP2 and CASP8, known as apoptosis initiators, were found to be significantly more abundant in SUP samples of transfected cells. Similarly, the executioner caspases CASP3, CASP6, and CASP7 were differentially expressed. While CASP6 was more highly expressed in control samples, CASP3 and CASP7 were more abundant in AAV-producing cells. Generally, elevated caspase levels could potentially result in reduced cell viability. Therefore, caspase levels might be used to monitor overall condition of the cell culture in order to maintain high levels of viability before harvest.

When considering improved AAV titers, especially endocytosis could be an interesting focus of future studies. Usually, recombinant AAV is purified from cell lysates. However, AAV is also released into the cell culture medium [28]. In terms of downstream processing, purifying AAV from cell culture medium might be beneficial due to the lower abundance of contaminating host cell proteins. Therefore, blocking endocytosis could result in higher AAV titers in the cell culture media. Similarly, internal processing of produced AAVs in lysosomes might result in proteasomal degradation, thus having a detrimental effect on AAV recovery. Hence, the use of proteasome inhibitors during production of AAVs could be explored.

**Figure 5:**
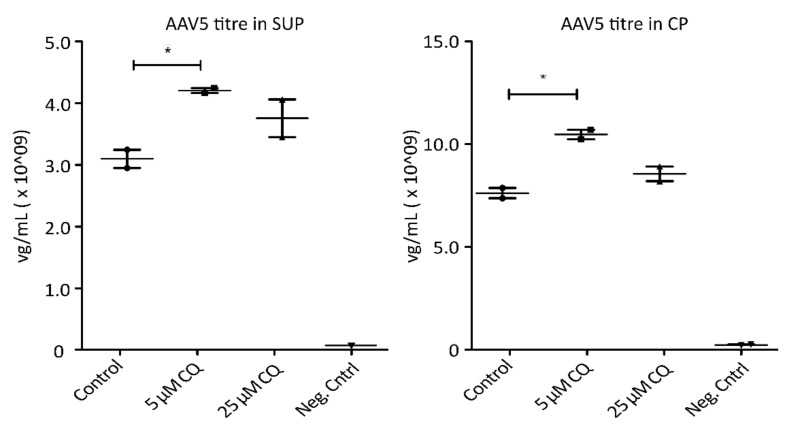
AAV5 concentration in viral genomes per mL (mg/mL) 72 h post-transfection following chloroquine (QC) treatment. Shown are titers measured via qPCR in cell culture media (SUP, **left**) as well cell pellets (CP, **right**). Significance was determined using a two-tailed t-test comparing treated samples vs. control (* *p* ≤ 0.05; n = 3).

To demonstrate the potential of proteomic profiling to enable knowledge-driven modifications of processing conditions, AAV5-expressing HEK293 cells were treated with 5 µM and 25 µM chloroquine (CQ), respectively. Chloroquine has been shown to reduce endocytosis as well as endosomal acidification [29,30,31]. As depicted in Figure 5, treatment of the cells with 5 µM CQ resulted in a significant increase of AAV titers in both, SUP and CP samples, without having a detrimental effect on cell viability. A higher concentration of AAV particles in cell culture media could be the result of reduced uptake by the host cells. A higher titer observed in cell lysates, on the other hand, could be the consequence of decreased proteasomal degradation of AAVs. Hence, treating cells with the correct, non-toxic concentration of CQ seems to prevent the pH drop required for viral release from endosomes, therefore trapping the particles until the cells are harvested and thereby have a beneficial effect on yield. While these are only preliminary results requiring further investigations, targeted modulation of endocytic processes due to findings during proteome analysis resulted in an increase of AAV titers by more than 35%. This clearly highlights the necessity and benefits of a better understanding of the cellular processes involved during recombinant AAV production. Overall, GO analysis revealed several interesting cellular processes which might be used to sustain ideal processing conditions during cell culture.

#### 3.2.2. Pathway Analysis

To assess whether certain protein expression levels result in activation or inhibition of corresponding cell signaling pathways, Qiagen’s IPA software was used. The resulting canonical pathways and their activation z-scores are listed in Table 2. Shown are the top 15 most significant hits, based on *p*-values, for CP samples. A comprehensive list of canonical pathways can be found in the Supplementary Materials.

As indicated, EIF2 signaling, oxidative phosphorylation, and the spliceosomal cycle were significantly activated in AAV5-producing cells. EIF2 regulates translation, hence protein synthesis in eukaryotic cells. As recently summarized by Liu et al., EIF2 becomes activated upon phosphorylation, initiated as a stress response, for example, during viral infection [32]. Phosphorylation of EIF2 is regulated by four host cell kinases. While those kinases were not identified in the present study, activation of EIF2 signaling in the context of transfected cells for recombinant AAV production is to be expected. Whether the extent of activation correlates with productivity of the host cells remains to be elucidated.

Similarly, alternative splicing, a crucial step for mRNA translation, was found to be activated [33]. While this is generally a fundamental process in viable cells, higher activation of the spliceosomal cycle upon transfection of cells is expectable. Nevertheless, expression of AAV capsid proteins relies on alternate splicing [34], and efforts have been undertaken to optimize this process, resulting in various ratios of capsid proteins VP1, VP2, and VP3. This is of particular interest, as the VP ratio was found to correlate with infectivity of the produced AAV [35,36]. So far, only optimization of vector design has been exploited to modify VP ratios. However, looking into involved signaling in host cells might reveal further possibilities.

Another interesting observation is the activation of oxidative phosphorylation. As mentioned before, we have shown previously that increased recombinant protein productivity in CHO cells can be achieved upon a higher level of oxidative phosphorylation [5]. Cellular energy metabolism can easily be targeted by changes in the bioprocessing conditions. Therefore, increased titers might be possible via targeted optimization of cell culture conditions, aiming for elevated levels of pyruvate metabolism and oxidative phosphorylation.

Pathway analysis further revealed cellular processes that might be utilized to control PTM levels in rAAV. In this context, even though not found to be significant, the SUMOylation pathway was activated in transfected cells, which aligns with earlier findings [37]. Moreover, AAV2 was previously found to be SUMOylated during production, and modification levels correlated with AAV gene transduction [38].

In summary, pathway analysis showed several interesting cellular processes that are involved in maintaining cell viability, and translational processes were found to be activated in recombinant AAV5-producing HEK293 cells. Additionally, relevant pathways possibly affecting PTMs of the produced AAV were found.

## 4. Conclusions

With numerous ongoing clinical studies, AAV-based therapy of genetic diseases is on the rise. Existing transient triple transfection systems to produce AAV vectors for gene therapy are still inefficient and will require considerable improvement, particularly to ensure affordable treatments for larger disease indications. One of the first steps in achieving this is to gain a better understanding of the molecular mechanisms underpinning AAV synthesis in host production cell lines such as HEK293. Research within the biopharmaceutical industry has shown that detailed studies of host cell expression systems allow for targeted cell engineering, which has resulted in increased production titers.

Therefore, in the present study, proteome analysis of AAV5-producing HEK293 cells was performed. The obtained results revealed strong regulation of proteins involved in cell organization and biosynthesis. Furthermore, gene ontology and pathway analysis showed differential expression of endosomal-lysosomal proteins, an increase in translational processes, as well as an altered energy metabolism. Interestingly, SUMOylation and glycosylation pathways were observed to be affected upon transfection of the cells for AAV production. To demonstrate possible applications arising from the presented data, AAV-expressing cells were treated with chloroquine, aiming towards an alteration of endocytic processes. Thereby, titers in the cell culture media as well as in cell lysates were successfully increased by more than 35%.

In conclusion, while some of our findings might require further investigations, the data presented can be used for knowledge-driven improvements of recombinant AAV expression. Targeted modulation of certain signaling pathways could in the future allow for increased product titers with controlled levels of PTMs and for the maintenance of optimized processing conditions to sustain cell viability.

## Figures and Tables

**Table 1:** Cell count, viability, and cell size (µm) of HEK293 cells, as well as AAV5 concentration in viral genomes per mL (vg/mL) 72 h after transfection. N.d. = not detected.

Sample	Live Cells	Viability	Cell Size	AAV5 Titer	% GFP Positive Cells
Transfected 1	4.00 × 10^6^	93.50%	11.5	1.10 × 10^10^	61.9
Transfected 2	3.70 × 10^6^	94.40%	12.3	4.12 × 10^9^	61.7
Transfected 3	2.07 × 10^6^	91.20%	10.3	1.03 × 10^10^	65.7
Control 1	3.24 × 10^6^	98.50%	11.1	n.d.	n.d.
Control 2	2.88 × 10^6^	96.30%	9.2	n.d.	n.d.
Control 3	5.70 × 10^6^	98.30%	13.2	n.d.	n.d.

**Table 2:** Results of pathway analysis using IPA (QIAGEN Inc., https://www.qiagenbioinformatics.com/products/ingenuity-pathway-analysis, accessed on 20 April 2021). Shown are activation z-scores and –log *p*-values of the top 15 canonical pathways in cell pellet (CP) samples after transfection (n = 3). Z-scores ± 2 are considered significant. Positive z-scores indicate activation, while negative values show inhibition of the corresponding pathway.

	Ingenuity Canonical Pathways	–log (*p*-Value)	z-Score
Top 15 canonical pathways (*p*-value)	EIF2 Signaling	45.5	2.21
	Oxidative Phosphorylation	23	2.36
	Inhibition of ARE-Mediated mRNA Degradation Pathway	22.1	−1.66
	Regulation of eIF4 and p70S6K Signaling	21.9	−1.83
	mTOR Signaling	18.9	−1.66
	Spliceosomal Cycle	18.5	2.34
	Remodeling of Epithelial Adherens Junctions	16	−2.13
	Coronavirus Pathogenesis Pathway	13.3	−1.61
	Sumoylation Pathway	10	1.72
	Integrin Signaling	9.85	−2.24
	14-3-3-mediated Signaling	8.06	−2.29
	Ephrin Receptor Signaling	7.11	−1.83
	VEGF Signaling	7.01	−1.72
	Ferroptosis Signaling Pathway	6.7	−2.18

## Data Availability

Raw data was deposited to the ProteomeXchange Consortium via the PRIDE partner repository with the dataset identified PXD028154.

## Supplementary Materials

The following are available online at www.mdpi.com/1422-0067/222/11/1499/s1, Supplementary Data: Proteomics results.

## References

[1] Wang D., Tai P.W.L., Gao G. (2019). Adeno-associated virus vector as a platform for gene therapy delivery. Nat. Rev. Drug Discov..

[2] Zhao H., Lee K.J., Daris M., Lin Y., Wolfe T., Sheng J., Plewa C., Wang S., Meisen W.H. (2020). Creation of a High-Yield AAV Vector Production Platform in Suspension Cells Using a Design-of-Experiment Approach. Mol. Ther. Methods Clin. Dev..

[3] Joshi P.R.H., Cervera L., Ahmed I., Kondratov O., Zolotukhin S., Schrag J., Chahal P.S., Kamen A.A. (2019). Achieving High-Yield Production of Functional AAV5 Gene Delivery Vectors via Fedbatch in an Insect Cell-One Baculovirus System. Mol. Ther. Methods Clin. Dev..

[4] Walsh G. (2018). Biopharmaceutical benchmarks 2018. Nat. Biotechnol..

[5] Strasser L., Farrell A., Ho J.T.C., Scheffler K., Cook K., Pankert P., Mowlds P., Viner R., Karger B.L., Bones J. (2021). Proteomic Profiling of IgG1 Producing CHO Cells Using LC/LC-SPS-MS(3): The Effects of Bioprocessing Conditions on Productivity and Product Quality. Front. Bioeng. Biotechnol..

[6] Lee M.S., Kim K.W., Kim Y.H., Lee G.M. (2003). Proteome analysis of antibody-expressing CHO cells in response to hyperosmotic pressure. Biotechnol. Prog..

[7] Kunert R., Reinhart D. (2016). Advances in recombinant antibody manufacturing. Appl. Microbiol. Biotechnol..

[8] Farrell A., McLoughlin N., Milne J.J., Marison I.W., Bones J. (2014). Application of multi-omics techniques for bioprocess design and optimization in chinese hamster ovary cells. J. Proteome Res..

[9] Stolfa G., Smonskey M.T., Boniface R., Hachmann A.B., Gulde P., Joshi A.D., Pierce A.P., Jacobia S.J., Campbell A. (2018). CHO-Omics Review: The Impact of Current and Emerging Technologies on Chinese Hamster Ovary Based Bioproduction. Biotechnol. J..

[10] Dong B., Duan X., Chow H.Y., Chen L., Lu H., Wu W., Hauck B., Wright F., Kapranov P., Xiao W. (2014). Proteomics analysis of co-purifying cellular proteins associated with rAAV vectors. PLoS ONE.

[11] Aponte-Ubillus J.J., Barajas D., Sterling H., Aghajanirefah A., Bardliving C., Peltier J., Shamlou P., Roy M., Gold D. (2020). Proteome profiling and vector yield optimization in a recombinant adeno-associated virus-producing yeast model. Microbiologyopen.

[12] Lavado-Garcia J., Jorge I., Cervera L., Vazquez J., Godia F. (2020). Multiplexed Quantitative Proteomic Analysis of HEK293 Provides Insights into Molecular Changes Associated with the Cell Density Effect, Transient Transfection, and Virus-Like Particle Production. J. Proteome Res..

[13] Hughes C.S., Moggridge S., Muller T., Sorensen P.H., Morin G.B., Krijgsveld J. (2019). Single-pot, solid-phase-enhanced sample preparation for proteomics experiments. Nat. Protoc..

[14] Lafuente E.M., van Puijenbroek A.A., Krause M., Carman C.V., Freeman G.J., Berezovskaya A., Constantine E., Springer T.A., Gertler F.B., Boussiotis V.A. (2004). RIAM, an Ena/VASP and Profilin ligand, interacts with Rap1-GTP and mediates Rap1-induced adhesion. Dev. Cell.

[15] Lee S.H., Dominguez R. (2010). Regulation of actin cytoskeleton dynamics in cells. Mol. Cells.

[16] Berry G.E., Asokan A. (2016). Cellular transduction mechanisms of adeno-associated viral vectors. Curr. Opin. Virol..

[17] Rose D.R. (2012). Structure, mechanism and inhibition of Golgi alpha-mannosidase II. Curr. Opin. Struct. Biol..

[18] Mietzsch M., Broecker F., Reinhardt A., Seeberger P.H., Heilbronn R. (2014). Differential adeno-associated virus serotype-specific interaction patterns with synthetic heparins and other glycans. J. Virol..

[19] Aloor A., Zhang J., Gashash E.A., Parameswaran A., Chrzanowski M., Ma C., Diao Y., Wang P.G., Xiao W. (2018). Site-Specific N-Glycosylation on the AAV8 Capsid Protein. Viruses.

[20] Delobel A. (2021). Glycosylation of Therapeutic Proteins: A Critical Quality Attribute. Methods Mol. Biol..

[21] Willems E., Dedobbeleer M., Digregorio M., Lombard A., Lumapat P.N., Rogister B. (2018). The functional diversity of Aurora kinases: A comprehensive review. Cell Div..

[22] Dietmair S., Hodson M.P., Quek L.E., Timmins N.E., Gray P., Nielsen L.K. (2012). A multi-omics analysis of recombinant protein production in Hek293 cells. PLoS ONE.

[23] Xin Z., Pu L., Gao W., Wang Y., Wei J., Shi T., Yao Z., Guo C. (2017). Riboflavin deficiency induces a significant change in proteomic profiles in HepG2 cells. Sci. Rep..

[24] Mahalik S., Sharma A.K., Mukherjee K.J. (2014). Genome engineering for improved recombinant protein expression in Escherichia coli. Microb. Cell Fact..

[25] Suikkanen S., Antila M., Jaatinen A., Vihinen-Ranta M., Vuento M. (2003). Release of canine parvovirus from endocytic vesicles. Virology.

[26] Lins-Austin B., Patel S., Mietzsch M., Brooke D., Bennett A., Venkatakrishnan B., Van Vliet K., Smith A.N., Long J.R., McKenna R. (2020). Adeno-Associated Virus (AAV) Capsid Stability and Liposome Remodeling During Endo/Lysosomal pH Trafficking. Viruses.

[27] Tang D., Kang R., Berghe T.V., Vandenabeele P., Kroemer G. (2019). The molecular machinery of regulated cell death. Cell Res..

[28] Kimura T., Ferran B., Tsukahara Y., Shang Q., Desai S., Fedoce A., Pimentel D.R., Luptak I., Adachi T., Ido Y. (2019). Production of adeno-associated virus vectors for in vitro and in vivo applications. Sci. Rep..

[29] Al-Bari M.A.A. (2017). Targeting endosomal acidification by chloroquine analogs as a promising strategy for the treatment of emerging viral diseases. Pharmacol. Res. Perspect..

[30] Hu T.Y., Frieman M., Wolfram J. (2020). Insights from nanomedicine into chloroquine efficacy against COVID-19. Nat. Nanotechnol..

[31] Delvecchio R., Higa L.M., Pezzuto P., Valadao A.L., Garcez P.P., Monteiro F.L., Loiola E.C., Dias A.A., Silva F.J., Aliota M.T. (2016). Chloroquine, an Endocytosis Blocking Agent, Inhibits Zika Virus Infection in Different Cell Models. Viruses.

[32] Liu Y., Wang M., Cheng A., Yang Q., Wu Y., Jia R., Liu M., Zhu D., Chen S., Zhang S. (2020). The role of host eIF2alpha in viral infection. Virol. J..

[33] Kornblihtt A.R., Schor I.E., Allo M., Dujardin G., Petrillo E., Munoz M.J. (2013). Alternative splicing: A pivotal step between eukaryotic transcription and translation. Nat. Rev. Mol. Cell Biol..

[34] Trempe J.P., Carter B.J. (1988). Alternate mRNA splicing is required for synthesis of adeno-associated virus VP1 capsid protein. J. Virol..

[35] Bosma B., du Plessis F., Ehlert E., Nijmeijer B., de Haan M., Petry H., Lubelski J. (2018). Optimization of viral protein ratios for production of rAAV serotype 5 in the baculovirus system. Gene Ther..

[36] Wang Q., Wu Z., Zhang J., Firrman J., Wei H., Zhuang Z., Liu L., Miao L., Hu Y., Li D. (2017). A Robust System for Production of Superabundant VP1 Recombinant AAV Vectors. Mol. Ther. Methods Clin. Dev..

[37] Holscher C., Sonntag F., Henrich K., Chen Q., Beneke J., Matula P., Rohr K., Kaderali L., Beil N., Erfle H. (2015). The SUMOylation Pathway Restricts Gene Transduction by Adeno-Associated Viruses. PLoS Pathog..

[38] Chen Q., Njenga R., Leuchs B., Chiocca S., Kleinschmidt J., Muller M. (2020). SUMOylation Targets Adeno-associated Virus Capsids but Mainly Restricts Transduction by Cellular Mechanisms. J. Virol..

